# Cholera deaths in Soho, London, 1854: Risk Terrain Modeling for epidemiological investigations

**DOI:** 10.1371/journal.pone.0230725

**Published:** 2020-03-30

**Authors:** Joel M. Caplan, Leslie W. Kennedy, Christine H. Neudecker

**Affiliations:** Rutgers School of Criminal Justice, Rutgers University–Newark, Newark, New Jersey, United States of America; Montclair State University, UNITED STATES

## Abstract

Risk Terrain Modeling (RTM) is a spatial analysis technique used to diagnose environmental conditions that lead to hazardous outcomes. Originally developed for applications to violent crime analysis, RTM is utilized here to analyze Dr. John Snow’s data from the 1854 cholera outbreak in London to demonstrate its potential value to contemporary epidemiological investigations. Dr. Snow saved countless lives when he traced the source of the cholera outbreak to a specific water pump through inductive reasoning, which he communicated through maps and spatial evidence. His methods have since inspired several fields of scientific inquiry. Informed by the extant research on RTM, we speculated that it could have helped test Dr. Snow’s hypothesis about cholera and empirically identified the sole source of contaminated well water. We learned that it could and, although it was not available to Dr. Snow in the 1800s, we discuss RTM’s implications for present-day research and practice as it relates to the analysis, prevention and treatment of cholera and other public health threats around the world.

## Introduction

Dr. John Snow is frequently regarded as the father of epidemiology based on his storied success at curbing the cholera outbreak in the Soho neighbourhood of the Parish of Saint James, Westminster, London in 1854. He used primary data collection and crude mapping techniques as spatial evidence to trace the source of the epidemic to a specific well water pump. His hypothesis that cholera was a waterborne disease and his investigation to prove it informed decisions by local officials to decommission the offending well water pump, thereby stopping the spread of cholera. Prior to Dr. Snow’s investigation, cholera was thought to be an airborne disease [1]. As one can imagine, strategies to combat airborne diseases versus waterborne disease are very different. Further, eradication of a waterborne disease without finding the source is impossible and puts lives at continued risk of illness or death. Dr. Snow’s finding, then, both revolutionized the way cholera was understood and how disease source detection could be accomplished through epidemiological investigation. This paper is not the first to analyze Dr. Snow’s data through geographic information systems (GIS) mapping, or to offer empirical support for why Snow was wise to challenge medical theories that were popular at the time [2; 3; 4; 5]. However, this paper is a unique complement to prior studies by offering Risk Terrain Modeling (RTM) as a contemporary spatial diagnostic method that can be used to embark on similar epidemiological investigations. The historical significance of Dr. Snow’s work is a compelling reason to use it as a baseline for comparison purposes and spatial hypothesis testing with RTM.

RTM is a spatial analysis method primarily used to assess micro-level environmental risks of crime throughout a landscape. It was originally developed in the field of criminal justice and applied to the study of violent crimes, such as shootings [6; 7]. Researchers and practitioners in a variety of jurisdictions use RTM to diagnose environmental conditions that lead to crime outcomes. In its most traditional sense it has successfully been used by police and public safety agencies to analyze violent or property crimes [8; 9; 10; 11; 12; 13; 14; 15] and deploy resources to places in greatest need of intervention programs. Outside of crime applications, RTM has been applied in correctional settings dealing with a range of topics surrounding recidivism of parolees [16; 17], and to issues of terrorism and security [18; 19]. Other applications of RTM, perhaps most related to public health and our current research topic, involve matters of child abuse [20], domestic violence [21], residential evictions [22], homelessness [23], and traffic crashes [10; 24]. RTM is multifaceted and can be applied to a broad range of subject matters that have a spatial component.

In this paper we advance RTM to the field of epidemiology by using it to analyze Dr. Snow’s data from London’s 1854 cholera outbreak. Ultimately, we draw the same conclusion as Dr. Snow, and confirm his hypothesis that water pump number 1 was the culprit. The value in this is twofold. First, we show that using a spatial analysis approach such as RTM to diagnose environmental factors connected with disease outbreaks is a valid method of spatial analysis; Second, we demonstrate the advantages of using such a technique in discovering present-day disease sources in epidemic situations, or to identify vulnerable places and populations at the micro-level before outbreaks emerge. This paper continues with a general background of cholera before describing the methodology behind RTM. Results from the RTM analysis of Dr. Snow’s data are discussed next. New insights and broader implications of RTM for research and practice conclude this paper.

## Background

Cholera is a global threat to public health despite all the efforts to eradicate it over many centuries. Estimates show that there are millions of new cases of cholera yearly and that as many as 143,000 deaths could be attributed to this disease each year in 69 endemic countries [25]. The World Health Organization (WHO) [26] states that cholera is non-discriminatory, affecting or killing both children and adults within hours when left untreated after symptoms emerge following the ingestion of contaminated food or water [27]. If people are treated early, and the source of contamination is mitigated, a full recovery to people and populations can be expected in most situations. Therefore, rapidly finding the source of this virulent disease once outbreaks emerge is paramount to saving lives. Whereas common symptoms of cholera in people can appear as diarrhea or dehydration, the geo-spatial distributions of infected people within a particular area may be symptomatic of the source location of the disease. Such a spatial perspective [28] suggests that an outbreak among infected people within a particular area becomes the local environment’s *symptom* of a public health problem in need of diagnosis and upstream intervention.

Cholera is characterized as endemic. Endemic cholera areas are geographic locations wherein cholera has been detected and found to be locally transmitted within the last three years [26]. Local transmission means that it originated from within that area and was not brought there from outside the immediate perimeter, such as via importation of contaminated water. Whether cholera epidemics occur inside or outside of cholera endemic areas [26], the spread of cholera is often connected to known risk factors [4; 26; 29; 30; 31]–a summary of which can be found in Table 1. If we know the locations of these risk factors and their interaction affects, we could forecast where outbreaks are more likely to originate at the micro-spatial level.

**Table 1:** Summary of risk factors for cholera.

• **Limited or no access to clean water and proper sanitation facilities**
• **Refugee camps**
• **Slums**
• **Poor rural areas**
• **Bathing in rivers**
• **Residing a long distance from a water source**
• **Eating dried fish**
• **Shared latrine with 3 or more households**
• **A person’s time of arrival in refugee camp**
• **Proximity to surface water**
• **Areas of high population density**
• **Areas with high rates of poor educational level**

Drawing on work that has been done on crime contagion and hot spot formation, Caplan and Kennedy [7] explain that certain features of the landscape exert spatial influences on human behaviors that affect a place’s vulnerability to hazardous outcomes–which is why crimes emerge, cluster and persist over time. Whereas crimes cluster at particular places over time to form ‘crime hot spots’, it is likely that these spots exist because environmental risk factors that tie to these crimes also cluster [28]. Something similar may be said of disease contagion, whereby the environmental backcloth affects how place features relate to and influence human behavior patterns, situational contexts, localized outbreaks and then persistent epidemics. Interventions prescribed to treat people and places require an understanding of the environmental behavior settings and spatial risk factors present at the existing or potential contamination source(s) in order to sufficiently quell outbreaks or prevent their spread [26; 32].

While risk factors for endemic cholera cases can vary at both the macro and micro levels, just a few prevention strategies are generally seen as paramount to controlling the disease’s spread. These include access to clean water, physically covering water sources, proper sanitation facilities, and educational campaigns [26; 29; 30; 31]. So, while programs to effectively combat cholera outbreaks already exist and have proven reliable around the world, analytical tools to inform resource deployments and interventions at the micro level could further improve public health impacts. Dr. Snow’s own investigation methods of mapping cholera deaths and water pumps led to pump 1 being decommissioned, i.e., the physical covering of an offending water source. But while he suspected contaminated water to be causing or contributing to the 1854 Soho cholera outbreak, we must recognize that his hypothesis was revolutionary and evolutionary at the time. Snow’s analytical methods helped to shape the evolutionary state of knowledge about cholera. Research literature generally suggests that risk factors for cholera aggravate one another when they are located at the same places, which create spatial vulnerabilities to disease outbreaks. But within these settings and groups of people who live there, techniques to zero-in on particular locations of contamination sources of an emergent outbreak at the micro level is understudied. There are no known applications of RTM to study cholera, yet RTM excels at diagnosing environmental risk factors and their interactions at the micro-level to create vulnerable places.

Particularly within the context of treatment and prevention, an analysis such as RTM to inform targeted resource deployments and actions for cholera control and risk management is imperative [26]. A global three-part strategy for combatting cholera was proposed by the Global Task Force on Cholera Control (GTFCC) [32] and endorsed in May of 2018 at the 71^st^ World Health Assembly [26; 33]. The first part of GTFCC’s [32] strategy is “early detection and quick response to contain outbreaks” [32, p. 3] because if cholera is detected in its early stages, individuals are more likely to survive. The second part is a “targeted multi-sectoral approach to prevent cholera recurrence” [32, p. 3] because eliminating cholera requires careful observance of the disease and risk factors associated with it. Finally, the strategy calls for “[a]n effective mechanism of coordination for technical support, advocacy, resource mobilization, and partnership at local and global levels” [32, p. 3]. Validating RTM with Dr. Snow’s well-known and lauded case study of cholera control offers an important first step in demonstrating RTM’s relevance to the GTFCC mission and other contemporary epidemiological issues.

There are likely many environmental features of the built environment that existed in Soho where the Cholera outbreak emerged in 1854. Dr. Snow was specifically interested in well water pumps because he hypothesized that Cholera was transmitted via water. The notations of water pumps in his original maps speaks to this hypothesis, and suggests these maps were an effort to explore this connection further. In this regard, our study starts from the premise that Snow had a hypothesis in need of testing and we apply RTM as a contemporary spatial method for hypothesis testing. We wondered if RTM was available in the mid-1800s, could it have helped shape Dr. Snow’s risk narrative for Cholera and identify the sole source of contaminated water? Yes, it can. We explain how in the next section.

## Methods

### Risk Terrain Modeling

RTM has three key processes: (1) standardizing disparate data sets to a common geography, (2) diagnosing spatial risk factors, and (3) articulating spatial vulnerabilities [7]. Caplan and Kennedy [7] outline 10 steps required to achieve the relative risk values associated with a study area for the problem of interest. The first step is to choose an outcome event. Cholera is the outcome event for this study. Second, the researcher must determine the study area they wish to examine. Here, our study area is the Soho area of London. Third, a time period must be selected. Our time period is 1854 because that was when the cholera outbreak was recorded. The fourth step is to identify potential risk factors that are available for testing in our analysis. While many potential risk factors can be selected as inputs for testing with RTM [28], ‘water pumps’ was the only risk factor category tested in the cholera case study. Further, while Dr. Snow hypothesized that water pumps, among all other possible environmental features (i.e., potential risk factors), were connected to cholera, he did not know which of the many water pumps was the culprit. Therefore, we also treated each of the 8 well water pumps located in the study area as individual inputs to create 8 potential “risk factors”. This allowed for spatial hypothesis testing with RTM that also zeroed-in on the likely problematic well water pump. The fifth step requires spatial data to be obtained that is representative of the time period under study. Here, these would be the geographic locations of individual cholera victims and well water pumps, as well as a map of the study area boundary representative of the 1854 time period. These data were obtained from an online source that digitized John Snow’s original data [34].

Steps six through nine require GIS mapping and statistical analyses that are automated by the RTMDx software, which we obtained from the Rutgers University Center on Public Security (see www.rtmdx.gratis for free access). These steps involve mapping the spatial influences of potential risk factors and then statistically selecting and weighting the significant factors to include in a final regression model. In step six, we set the parameters for testing spatial influences of risk factors within RTMDx as a function of ‘proximity’, which models Euclidean distances between the risk factor features (water pumps) and topic issue incidents (cholera victims) at multiple bandwidths.

Steps seven and eight, respectively, are to empirically select and weight risk factors [7]. This involves cross-validation of risk factor inputs to minimize the possibility of spuriousness when adding a lot of risk factors into a model all-at-once. This results in a smaller set of risk factors (i.e. variables) with non-zero coefficients that are then entered into a bidirectional step-wise regression process that considers both Poisson and Negative Binomial distributions. Bayesian Information Criteria (BIC) scores are compared at each step until there is no improvement in the BIC score and only one ‘best’ regression model is selected. Relative risk values (RRV) of the significant risk factors in the model are created from the exponentiation of the coefficients from the regression. Step nine combines the model factors in a GIS to produce a final risk terrain map [7]. RTMDx outputs are tabular and cartographic; for each significant risk factor, tabular outputs include a relative risk value (RRV) and the optimal operationalization and distal (i.e., proximate) extent of spatial influence. The risk terrain map is produced with relative risk scores (RRSs) assigned to each micro place to convey the full range of relative spatial risks throughout the study area. See Caplan and Kennedy [7] and Caplan, Kennedy, and Piza, [35] for a more detailed discussion about the RTM process and statistical methods performed by RTMDx.

The tenth step is to interpret the analyses. This requires analysts to build a risk narrative around the key findings. A risk narrative is a data-informed story, or contextualized understanding, of how the findings relate to the dynamic interactions of people at places in the study setting. For example, in one application of RTM to gun violence in Atlantic City, New Jersey, police commanders created a risk narrative [28] for convenience stores, laundromats and vacant properties that were identified as top risk factors: They hypothesized that shootings were connected to drug sales and related turf conflicts whereby ‘convenience stores’ are the places where drug buyers are solicited; Nearby ‘Laundromats’ are locations where drug transactions are made; ‘vacant buildings’ located nearby are used by drug dealers as stash houses for drugs and weapons, or by drug buyers to use drugs after purchase. When police used this data-driven evidence from RTM to surmise that drugs, retail businesses, and vacant properties were related in these ways to the crime problem under study, they were able to build consensus that certain places will probably experience these crime incidents in the future. They had a shared narrative as to *why* crimes might persist at these places, which served as a catalyst for risk mitigation efforts coordinated among multiple stakeholders [36].

### RTM, cholera, and John Snow

A key strength of RTM is its ability to identify and weigh environmental features that are significantly correlated with the spatial patterns of outcome events of interest, such as cholera deaths. Dr. Snow suspected cholera to be a waterborne disease and hypothesized well water pumps to be the mechanism of transmission to people. He manually surveyed and mapped the locations of cholera deaths and used these patterns to deduce a contamination source and remove the water pump handle. But, could RTM have diagnosed this spatial connection if Dr. Snow couldn’t? We ran two separate risk terrain models using the water pump locations and addresses of the cholera victims in the Soho study area, as originally recorded by Dr. Snow. The first model (Model 1 in Table 2) treated all of the pumps as a single category, or one potential risk factor (variable), labeled “All Pumps”. The second model (Model 2 in Table 2) treated each of the 8 pumps as separate potential risk factors (variables), labeled “Pump 1” through “Pump 8”.

**Table 2:** Statistical results of Risk Terrain Models.

MODEL	SIGNIFICANT RISK FACTOR	OPERATIONALIZATION	SPATIAL INFLUENCE (METERS)	STANDARD ERROR	T-VALUE	COEFFICIENT	RELATIVE RISK VALUE (RRV)
1	All Pumps	Proximity	300	1.01	2.33	2.35	10.52
2	Pump 1	Proximity	200	0.31	10.89	3.38	29.34

## Results

**Fig 1:**
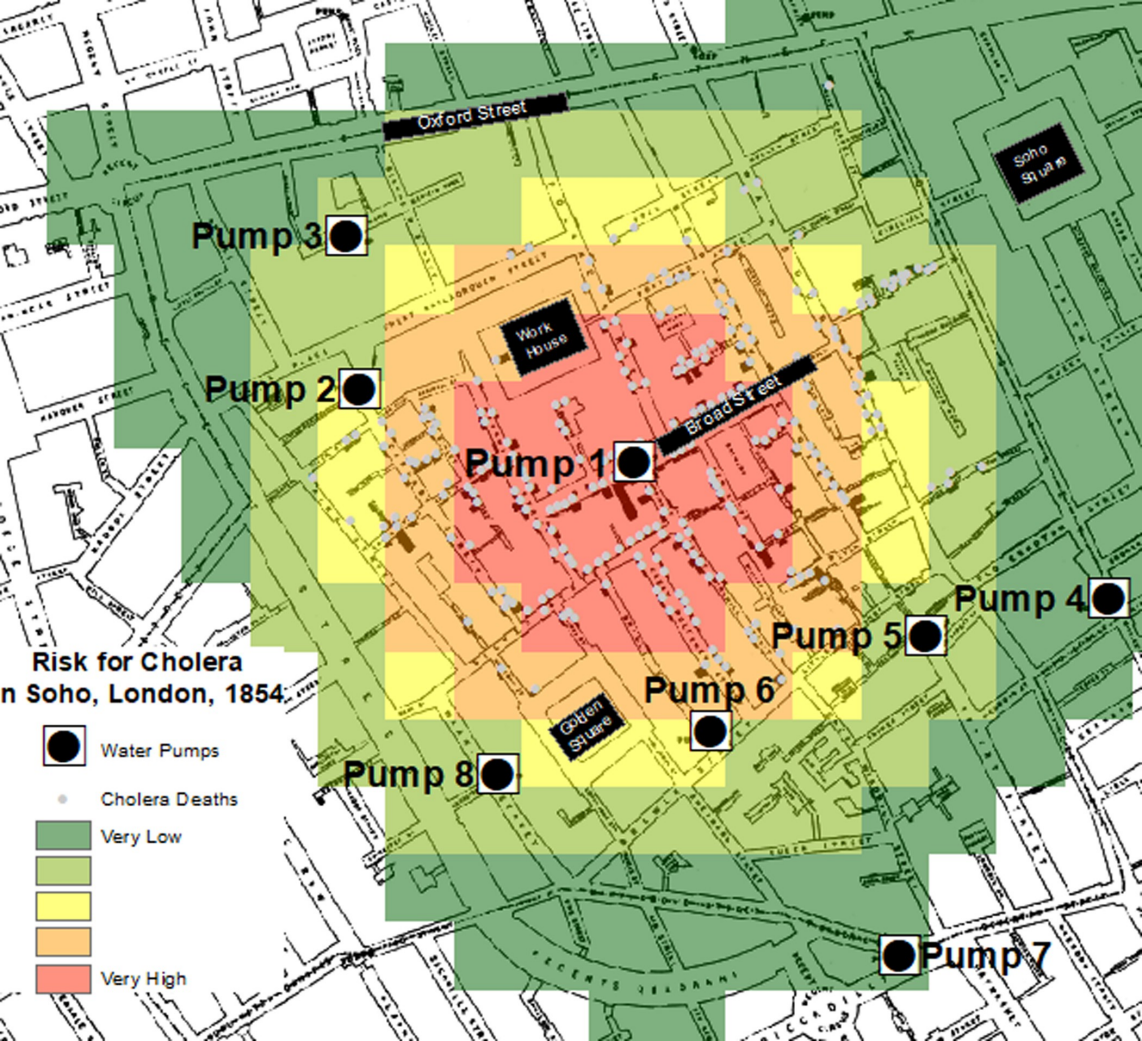
Risk Terrain Map of Model 2: High-risk places for cholera victimization in Soho, London, 1854. Map Sources: [37; 38].

RTM found significant spatial correlations between water pumps and cholera victims’ residences, suggesting that “water pumps” were a risk factor for cholera in the Soho Neighborhood of London in 1854. As shown in Table 2, residing within 300 meters from a water pump increased the likelihood of contracting cholera by a factor of 10.5. Of course, this finding is expected, and has face validity, given what we know today about Dr. Snow’s epidemiological case study. But we also know from history that one particular water pump within the aggregate category of “All Pumps” caused the infections. RTM identified this specific contamination source in the Model 2 analysis. As shown in Table 2, results from this analysis identified Pump 1 to be the only spatially significant risk factor out of all possible options; the other seven pumps were statistically excluded during the modeling process. Pump 1 was, in fact, the actual culprit in the 1854 cholera epidemic. According to the risk terrain model results in Table 2, residences within 200 meters from Pump 1 were over 29 times more likely to have cholera victims than residences at other places or near other pumps in the study area. Fig 1 shows the risk terrain map for Model 2, with the highest-risk area focused around Pump 1 and lower levels of risk at all other places throughout the study area.

## Discussion/conclusion

Dr. John Snow exemplified the value of mapping and scientific inquiry for epidemiology and public health when he investigated the 19^th^ century cholera outbreak in the Soho neighbourhood of London. His hypothesis that cholera was waterborne and his knowledge that water pumps spread and perpetuated the cholera outbreak was not a solution to the problem in-and-of-itself. It was his ‘risk narrative’ about how these pumps permitted the delivery of contaminated well water for drinking (that is, the situational context for disease transmission) that led him (or city officials) to remove the handle from Pump 1 as risk reduction strategy. Without a handle people could not drink water from the contaminated well and the threat was mitigated. Pump 1 did not spread the cholera by its mere physical presence; you had to ingest its water to become infected. So, the 1854 cholera outbreak in Soho had a simple risk narrative, validated and informed by the RTM analysis: More often than not, people are likely to use and drink water from the pump closest to their house. To disrupt this narrative and mitigate risk, Snow removed the water pump’s handle, thereby preventing anyone from getting water from the contaminated well, even if its location was the most convenient for some people. The data-informed risk narrative for how water Pump 1 connected to cholera victim locations led to a successful intervention strategy by Dr. Snow.

Dr. John Snow was an epidemiologist who explored the spatial interactions of people with places in relation to cholera. He collected geo-located data to create a spatial risk model and found the source of the cholera outbreak in 1854. Our RTM analysis was successful at confirming the spatial correlation between cholera victims and water pumps. Most importantly, RTM accurately identified Pump 1 out of all other options as the sole source of the deadly outbreak. Using Dr. Snow’s historically well-known survey data and accurate conclusion as a comparison threshold, this study demonstrated that RTM is a valid method for diagnosing environmental factors connected with disease outbreaks. This has many implications for research and practice today.

RTM could be used to combat contemporary cholera outbreaks around the world. For instance, it fits well within the GTFCC’s [32] multipart strategy for targeting the disease. RTM provides actionable information for early detection of contamination sources for quick responses at the micro level, and for pre-emptive actions at other vulnerable locations, which part one of the GTFCC strategy calls for. RTM complements the typical hot spot approach to assessment by adding *why* to the *where* [28; 8; 12; 35]. Hot spot maps depict historical clustering of the outcome events over time [36]. But within these areas, focusing on spatial risk factors at the highest-risk places (as identified by RTM) could potentially yield the best results for containing outbreaks quickly. Hot spot mapping shows where incidents have occurred but does not consider the spatial factors that make these areas opportunistic in the first place. They are merely signs or symptoms of environments that are highly suitable for problems to spread and, compared to RTM, they offer fewer insights for solutions to mitigate the spatial factors that aggravate these risks. RTM provides a spatial diagnosis that can be used for deliberate collaboration among multiple stakeholders (e.g., government, non-governmental/non-profit organizations, and private companies) and sectors (e.g., health, economy, welfare) to jointly prevent recurrence, as the second part of the GTFCC strategy calls for.

For instance, returning to the Atlantic City RTM and risk narrative for gun violence, this data driven evidence led to multi-stakeholder conversations about how to effectively target and remediate the problems at these locations. The police department sought to intervene by focusing preemptively on the risky places: Areas around laundromats received directed police patrols, and police officer ‘meet-and-greets’ with convenience store managers were implemented at frequent intervals each day. The city’s Planning and Development Department prioritized remediation of vacant properties, with the help of the neighborhood association members. And installations of new LED street lights (to replace dimmer halogen lamps) at the highest risk places was undertaken by the utility authority, in collaboration with city officials. In a similarly way, future studies of cholera may wish to apply traditional hot spot mapping to zero-in to areas of concentrated disease outbreaks, then add RTM analysis to identify the most vulnerable places within the hot zones that could likely be the disease source aggravating the risks of transmission. Upstream approaches for intervention by multiple stakeholders at the local and global levels could be used in tactical fashion to address the underlying causes of the problem at hand in order to suppress the outbreak and mitigate future risks. In this way, RTM could complement mechanisms of coordination for advocacy, resource mobilization and multilevel partnerships for treatment and prevention of cholera, which the third part of GTFCC’s strategy calls for.

Future research with RTM may be able to model cholera better than before because of the increasingly accessible public data sources that are available to measure potential environmental risk factors. Recall that one of RTM’s strengths is its ability to weigh multiple risk factors and identify the most significant ones. Table 1 lists several potential risk factors that could be used for spatially diagnosing cholera within cities or regions of nation-states around the world. Digital datasets of these various risk factors, such as rivers, streams, water towers, refugee camps, population density, etc. are increasingly available for download from open data portals and can permit actionable RTM analysis at a variety of geographic extents. Some modern risk factors might not have existed or been relevant to the Dr. Snow case study and, similarly, some factors might not relate to all areas of the world in the present-day. Generally, though, exploratory spatial analysis with RTM should consider all features of the study area and time period [7]. Easy access to modern datasets means that RTM can be readily used by epidemiologists and other first responders to efficiently allocate limited resources to the people and places that need them most. And this need not be limited to cholera cases.

Other diseases with similar sources for transmission as cholera, such as typhoid or dysentery, might also be combatted in data-informed ways using RTM. According to WHO, approximately three-quarters of all human disease is waterborne [39]. Generally, this is not an issue in industrialized countries since the water is cleaned and treated, however no country is completely immune. Take for example, the 1993 case in Milwaukee where 54 people were killed by water contaminated with cryptosporidium parvum [40]. In a second example, a study in the Bronx, New York City found people living within a ¼ mile from auto body painting shops, electro-planting firms, waste transfer stations or factories; or 150m from highways or truck routes were up to 66 percent more likely to be hospitalized for asthma [41]. Layer all of these factors together spatially, and you can begin to see how RTM might come into play for matters of asthma, housing or healthcare spending.

While RTM appears to be worth exploring further in a variety of sectors, it is important to note some limitations. RTM should only be used with reliable data sources. Analysts should strive to obtain as much reliable and valid incident data as is possible. On-the-ground primary data collection, as is often done early to verify and back trace outbreaks after they become known, remains important. It is equally important to do an exhaustive literature review and related data collection of known (potential) environmental risk factors of the disease (or other problem) under study. For example, a survey of well water pump locations would have been required here if secondary or administrative datasets of these landscape features were not already available. Open data portals make landscape feature datasets increasingly easy to get for many cities in the United States. But, primary data collection, crowdsourcing, or satellite imagery, to name a few options, may also be needed to produce data inputs for analysis.

RTM for disease outbreaks (control, prevention and risk management) is only useful if the human elements of skilled decision makers and first responders are available to make the spatial intel actionable. RTM should be considered as a supplement to existing methods, but never as a replacement or substitute to dwindling resources. In the context of these limitations, RTM appears to offer important insights for researchers and practitioners of public health, epidemiology and population studies.

## Supporting information

S1 File (ZIP)
