# Supplementary material for: Cholera deaths in Soho, London, 1854: Risk Terrain Modeling for epidemiological investigations
Source: PLoS One. 2020 Mar 30;15(3):e0230725. doi: 10.1371/journal.pone.0230725 (PMC7105112; doi:10.1371/journal.pone.0230725)
Supplement: S1 File — (ZIP) [file pone.0230725.s001.zip › Snow_London/RTM_Output/AllPumps_100m50m.zip!/AllPumps_100m50m/AllPumps_100m50m-report.html]

RTMDx Report


#### Result Summary

#### Analysis Input Details

#### Analysis Parameters

The Utility was provided with the following risk factors and parameters:

#### "Best" Model Specification

**R Text Summary**

#### Risk Terrain Map Production
